# Ramadan Fasting Leads to Shifts in Human Gut Microbiota Structured by Dietary Composition

**DOI:** 10.3389/fmicb.2021.642999

**Published:** 2021-02-18

**Authors:** Ikram Ali, Ke Liu, Danfeng Long, Shah Faisal, Mian Gul Hilal, Izhar Ali, Xiaodan Huang, Ruijun Long

**Affiliations:** ^1^School of Life Sciences, Lanzhou University, Lanzhou, China; ^2^School of Public Health, Lanzhou University, Lanzhou, China; ^3^Henan Provincial Center for Disease Control and Prevention, Zhengzhou, China

**Keywords:** ethnicity, Ramadan fasting, dietary habit, gut microbial diversity, MiSeq

## Abstract

The structure and diversity of human gut microbiota are directly related to diet, though less is known about the influences of ethnicity and diet-related behaviors, such as fasting (intermittent caloric restriction). In this study, we investigated whether fasting for Ramadan altered the microbiota in Chinese and Pakistani individuals. Using high-throughput 16S rRNA gene sequencing and self-reported dietary intake surveys, we determined that both the microbiota and dietary composition were significantly different with little overlap between ethnic groups. Principal Coordinate Analyses (PCoA) comparison of samples collected from both groups before and after fasting showed partial separation of microbiota related to fasting in the Pakistani group, but not in the Chinese group. Measurement of alpha diversity showed that Ramadan fasting significantly altered the coverage and ACE indices among Chinese subjects, but otherwise incurred no changes among either group. Specifically, *Prevotella* and *Faecalibacterium* drove predominance of Bacteroidetes and Firmicutes in the Pakistani group, while *Bacteroides* (phylum Bacteroidetes) were the most prevalent among Chinese participants both before and after fasting. We observed significant enrichment of some specific taxa and depletion of others in individuals of both populations, suggesting that fasting could affect beta diversity. Notably, *Dorea, Klebsiella*, and *Faecalibacterium* were more abundant in the Chinese group after fasting, while *Sutterella, Parabacteroides*, and *Alistipes* were significantly enriched after fasting in the Pakistani group. Evaluation of the combined groups showed that genera *Coprococcus, Clostridium_XlV*, and *Lachnospiracea* were all significantly decreased after fasting. Analysis of food intake and macronutrient energy sources showed that fat-derived energy was positively associated with *Oscillibacter* and *Prevotella*, but negatively associated with *Bacteroides.* In addition, the consumption of sweets was significantly positively correlated with the prevalence of *Akkermansia.* Our study indicated that diet was the most significant influence on microbiota, and correlated with ethnic groups, while fasting led to enrichment of specific bacterial taxa in some individuals. Given the dearth of understanding about the impacts of fasting on microbiota, our results provide valuable inroads for future study aimed at novel, personalized, behavior-based treatments targeting specific gut microbes for prevention or treatment of digestive disorders.

## Introduction

The human gut is populated by trillions of microbes which collectively work as a “hidden organ” (Harsch and Konturek, 2018), the composition and structure of which can shift dynamically under the influence of dietary changes, environment, genetics, medication, and lifestyle (Findley et al., 2016; Deschasaux et al., 2018). In addition, several previous studies have identified differences in microbiota profiles across various ethnic groups, potentially driven by host genetics, geographic separation, and markedly distinct lifestyles (Goodrich et al., 2014; Gupta et al., 2017). Yatsunenko et al. (2012) also found that the composition of fecal microbiota differed significantly among United States residents, Amerindian, and Malawian individuals from geographically separated locations, primarily due to distinct lifestyle habits and the contents of their diet.

In fact, diet is well-established as the main factor affecting microbiota structure by enriching for specific species and their metabolic functions (Claesson et al., 2012; Kolodziejczyk et al., 2019). For instance, cultures such as the Hadza people (hunter-gatherers in Peru) consume mostly raw or wild foods and, as a result, show notably higher gut microbe diversity than that observed in Western Urban populations (Obregon-Tito et al., 2015). Typically, rural populations consume greater proportions of foods containing a high fiber content, thus promoting the enrichment of microbial taxa such as Bacteroidetes (e.g., *Xylanibacter* and *Prevotella*) and reducing the prevalence of other, such as Firmicutes (De Filippo et al., 2010). Studies in humans have indicated that variations in dietary macro-nutrients, i.e., proteins, fats, and carbohydrates (CHO), can significantly affect the shape of their gut microbiota (Wu et al., 2011; David et al., 2014), while other study have suggested diet-related behaviors, such as fasting for religious purposes, can also contribute to the structure of gut microbiota (Ozkul et al., 2020).

As a widely practiced diet-related behavior, fasting is defined as partial or complete abstention from food intake. Ramadan fasting is a religious practice where practitioners of the Muslim faith undergo a yearly ritual in which no food or beverages are consumed between sunrise to sunset for 29–30 days (Aybak, 1996). In mice given low-fat or high-fat diets, a dietary schedule in which food intake is decreased can trigger changes in microbiota structure as well as urinary and serum metabolic profiles (Zhang et al., 2013). Furthermore, in humans, short-term (28 days) restriction of CHO can result in decreased numbers of butyrate-producing bacteria, as well as butyrate (Duncan et al., 2007). Another study found that calorie restriction over a 10 weeks period can lead to variations in microbiome composition, including enrichment for *Bacteroides* and decreased abundance of *Blautiacoccoides* (Santacruz et al., 2009). By contrast, extended caloric restriction (for 12 months) resulted in decreased Actinobacteria and increased Bacteroidetes content in feces (Ruiz et al., 2017).

Based on these reports, we examined the contributions of three factors, including fasting (diet-related behavior), ethnicity, and dietary intake on the structure and diversity of human gut microbiota. We hypothesized that, given a preponderance of evidence showing that diet is the primary driver of microbiota structure, fasting (intermittent caloric restriction) can negatively or positively affect the abundance specific taxa within the gut microbial community, in a manner potentially dependent on ethnicity. To test whether fasting thus deferentially affect the shape of gut microbiota in individuals of different ethnic backgrounds, we used high-throughput metagenomic sequencing and dietary surveys to evaluate the influence of fasting during Ramadan on fecal microbial diversity in two ethnic groups (Pakistani and Chinese). The study participants were not geographically separated and were demographically similar.

## Materials and Methods

### Ethics Agreement, Approval to Join, and Recruitment

All procedures were done under the Medical Ethics Committee approval of the School of Public Health (GW-20171013). Detailed information forms of the survey were signed by all participants before sampling.

The inclusion criteria for participants were living in close regional proximity, practiced fasting, and ranged in age from 18 to 40 years old, furthermore, participants were psychologically and physically healthy. We excluded volunteers in case of (1) diseases, including gastrointestinal diseases, chronic diseases, anorexia nervosa, cachexia, (2) insufficiency of liver and kidney, (3) smoking, (4) drinking alcohol, (5) the use of antibiotics within last 3 months (Mesnage et al., 2019). According to our inclusion and exclusion criteria, 34 healthy adult participants, living in the city of Lanzhou and distributed across two ethnic groups (16 Chinese and 18 Pakistani) were recruited. All participants have attended Ramadan fasting from May 15, 2018, to June 15, 2018; both survey and fecal samples, before and after Ramadan fasting, were collected from the two groups. As a result, participants were distributed into six groups as (i) Chinese before fasting (CBF), (ii) Pakistani before fasting (PBF), (iii) Chinese after fasting (CAF), (iv) Pakistani after fasting (PAF), (v) total before fasting (TBF), and (vi) total after fasting (TAF). Comparisons across ethnic groups were categorized into ethnic groups before fasting (CBF vs. PBF) and ethnic groups after fasting (CAF vs. PAF); while fasting groups were compared among Chinese groups (CBF vs. CAF), Pakistan groups (PBF vs. PAF), and the total subject (TBF vs. TAF).

### Survey and Dietary Information

A questionnaire was established to acquire personal and other measurement information, such as family background and dietary habits, to further collect individualized data. As previously reported, a 3 days 24-h food dietary recall was defined for the consumption of total foods and beverages (Schröder et al., 2001). Selected foods included vegetables, grains, poultry meat, livestock meat, leaf vegetables, eggs, seafood, beans, fruits, dairy, nuts, milk, and sweets. This questionnaire was also used to assess the dietary record of the last 6 months for each individual. Participant background, age, gender, dietary habits and antibiotic usage were all noted before sampling. A software was developed by Peaking Union Medical college and West China Centre of Medical sciences (CDGSS3.0), to be used for nutritional analysis of each subject’s total energy and nutrition. Principal coordinate analysis (PCoA) was used to measure the beta diversity of food components in the diet of each ethnic group by R software (version 3.2.1).

### Material Collection and DNA Extraction

Fecal samples were collected for all participants on the same day. Sample collection before fasting was conducted on the morning of May 15, 2018, while samples after fasting were retrieved on the morning of June 15, 2018. Samples were frozen in liquid nitrogen immediately upon receipt to maintain sample stability (regardless of collection date/time). Samples were further stored at −70°C for future experiments.

DNA extraction was performed according to the manufacturer’s instructions (QIAamp DNA Stool Mini Kit, QIAGEN, Hilden, Germany). An ND-2000 Nanodrop spectrophotometer (Thermo Fisher Scientific, Waltham, MA, United States) develop by the United States, was used to determine the DNA content. Electrophoresis on 1% agarose gel was performed to evaluate the size and integrity of DNA. Samples of DNA were stored at −20°C for further analyses.

### Polymerase Chain Reaction (PCR) Analysis and High Throughput Sequencing

A library of the 16S metagenomic sequencing was prepared according to Illumina guidelines. Library construction and amplification of bacterial 16S rRNA gene were performed by metagenomic sequencing. Respective 16S primers (Forward: CCTACGGGNGGCWGCAG; Reverse: GACTACHVGGGTATCTAATCC) were designed for the amplification of bacterial 16S rRNA gene (V3 and V4 variable region). The Thermal Cycler (ABI2720, Thermo Fisher Scientific) was utilized for the amplification of HiFi HotStar ReadyMix (2× KAPA), final reaction (25μl) includes microbial DNA using 2.5 μl of a template (5 ng/μl), 5 μl of respective primers (1 μM each), and 12.5 μl. The cycling parameters were as the following: (i) initial denaturation at 95°C for 3 min, (ii) 25 cycles of denaturation at 95°C for 30 s, annealing at 55°C for 30 s, and elongation at 72°C for 30 s, and (iii) final extension at 72°C for 5 min. The final PCR products were quantified with an Agilent 2100 Bioanalyzer (Agilent Technologies, Santa Clara, California, United States) and then purified according to the manufacturer’s protocol (AMPure XP beads, Beckman Coulter, Coulter, Brea, California, United States). DNA sequencing was conducted by the use of MiSeq Reagent Kit v3 (Illumina, San Diego, CA, United States). Raw reads were loaded into the European Nucleotide Archive under the succession number PRJEB38231^1^.

### Bioinformatics and Statistical Analysis

Sequencing data were submitted to quality control and bioinformatics analysis accuracy. The original off-machine data filtering was improved to obtain high-quality data. For this, the software TrimGalore of (version 0.4.2) was used to remove terminal sequences below 20 bp, adapter sequences, and those shorter than 100 bp. FLASH2 software was also used to merge sequences from the splicing of paired-end sequences. Moreover, Mothur (version 1.41.1) was used to find and remove primer sequences. Lastly, USEARCH software (version 10.0) was utilized to remove sequences with less than 100 bp and an error rate higher than 2. Representative sequences were classified according to the Database Project of Ribosome (Cole et al., 2009). Upon improving data quality, more than 97% of similar clusters were added into operational taxonomic units (OTUs). Mothur software was again used for alpha diversity analysis (including Chao1, observed species, ACE, Shannon, Simpson, and coverage) among groups. To assess the beta diversity, principal coordinate analysis (PCoA) based on Bray-Curtis distances was performed by R software (version 3.2.1). VENN analyses and rarefaction curves were calculated at the OTU level using the R (version 3.2.1) software (Colwell et al., 2012). Significant species among the different groups were observed by Linear discriminate analysis effect size (LEfSe) (Segata et al., 2011).

Statistical analysis was performed on alpha diversity, dietary, and taxonomic data. The Wilcoxon signed-rank test was applied to “before vs. after Ramadan fasting” comparisons (CBF vs. CAF, PBF vs. PAF, TBF vs. TAF) and the Mann-Whitney U test was applied to comparisons between ethnic groups (CBF vs. PBF, CAF vs. PAF). The R vegan package (permutations = 9,999) was used for permutational multivariate analysis of variance (PERMANOVA) and, therefore, to verify significant differences in the structure of gut microbiota (McArdle and Anderson, 2001). The R package was assessed to calculate the correlation of microbial genera with diet by Pearson’s correlation analysis. The SPSS version 23.0 was used for all statistical analyses.

## Results

### Dietary Habits May Change During Fasting in Different Groups

**FIGURE 1:**
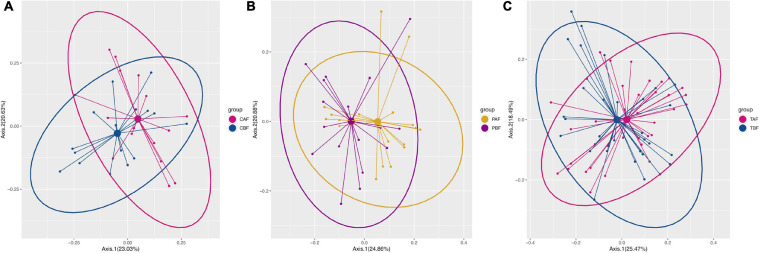
The nutrients intake profile by principal coordinate analysis (PCoA) of **(A)** Chinese before fasting vs. Chinese after fasting, **(B)** Pakistani before fasting vs. Pakistani after fasting, and **(C)** Total before fasting vs. Total after fasting groups. The variance presented by each component is written in brackets using Bray-Curtis.

PCoA analysis to identify differences in dietary profile revealed divergence among ethnic groups (CBF vs. PBF and CAF vs. PAF, Supplementary Figure S1), but considerable overlap in dietary intake within fasting groups (PBF vs. PAF, CBF vs. CAF, and TBF vs. TAF; Figure 1). The average daily food intake for fasting groups and the respective energy ratios and total energy provided by respective macro-nutrients (i.e., protein, CHO, and fat) are presented in Table 1. Within the Chinese fasting groups (Table 1), we observed a significant decrease in the intake of other vegetables and beans, whereas poultry intake increased after fasting (CBF vs. CAF). Within the Pakistani fasting group, grain intake and consumption of seafood, other vegetables, and vegetable supplements were all significantly increased after fasting (PBF vs. PAF). Regardless of ethnicity, the total population after fasting had a significantly higher poultry intake compared with their intake prior to fasting. The respective energy ratios showed that CHO provides the main source of energy, followed by fats, then proteins comprising the smallest proportion.

**TABLE 1:** Average daily food intake, total energy, and energy ratios provided by macronutrients across different Ramadan fasting groups.

**Food components**	**Chinese group**		**Pakistani group**		**Total group**	
	**CBF *n* = 16**	**CAF *n* = 16**	**PBF *n* = 18**	**PAF *n* = 18**	**TBF *n* = 34**	**TAF *n* = 34**
Grains intake (g)	735.01 (537.35–921.35)	791.67 (595.43–945.85)	580.35 (487.33–666.68)**	466.69 (333.35–533.35)	632.84 (511.51–783.51)	533.35 (400.02–779.17)
Milk intake (g)	83.33 (0.00–229.15)	83.33 (0.00–166.66)	151.71 (82.00–437.58)	161.66 (129.09–208.34)	126.66 (32.00–253.50)	130.02 (58.50–204.59)
Other vegetable intake (g)	136.65 (102.49–181.82)*	45.83 (10.83–113.33)	76.66 (74.20–207.92)	197 (132.91–332.48)**	116.66 (75.33–191.82)	132.50 (39.57–244.14)
Fruit intake (g)	0.00 (0.00–82.92)	0.00 (0.00–111.24)	103 (0.00–251.10)	216.16 (120.82–304.16)	20.00 (0.00–187.50)	119.99 (0.00–230.82)
Poultry intake (g)	0.00 (0.00–0.00)	8.33 (0.00–72.91)*	153.33 (79.16–244.99)	158.33 (83.33–354.15)	58.33 (0.00–159.16)	83.33 (0.00–166.66)
Livestock meat intake (g)	19.16 (10.11–87.83)	12.99 (0.58–66.99)	92.50 (41.67–127.09)	80.00 (41.25–127.49)	65.32 (16.91–105.83)	62.66 (6.91–104.99)
Condiments intake (g)	27.00 (27.00–189.49)	30.33 (27.00–272.82)	56.00 (55.75–163.34)	55.00 (55.00–109.99)	56.00 (27.00–163.34)	55.00 (27.37–247.84)
Egg intake (g)	14.67 (0.00–45.34)	45.34 (0.00–65.00)	31.83 (0.00–65.67)	0.00 (0.00–65.33)	21.83 (0.00–45.34)	19.33 (0.00–65.33)
Soyabeans intake (g)	106.66 (27.91–226.67)**	12.50 (0.00–33.34)	0.00 (0.00–0.00)	0.00 (0.00–0.00)	0.00 (0.00–98.33)*	0.00 (0.00–18.75)
Sweets and cake intake (g)	13.33 (0.00–36.45	0.00 (0.00–19.16)	24.33 (6.00–43.50)	30.00 (18.00–36.00)	20.33 (0.00–38.62)	19.00 (0.00–34.50)
Leaf vegetables intake (g)	16.67 (0.00–43.64)	30.00 (0.00–87.08)	0.00 (0.00–0.00)	0.00 (0.00–24.00)*	0.00 (0.00–16.67)	8.00 (0.00–65.74)*
Seafood (g)	0.00 (0.00–0.00)	0.00 (0.00–0.00)	0.00 (0.00–0.00)	0.00 (0.00–133.34)*	0.00 (0.00–0.00)	0.00 (0.00–5.00)*
Nuts intake (g)	0.00 (0.00–0.00)	0.00 (0.00–0.00)	0.00 (0.00–0.00)	0.00 (0.00–0.00)	0.00 (0.00–0.00)	0.00 (0.00–0.00)
**Total energy and energy ratios provided by macronutrients**						
Energy (kcal)	2400.70 (2025.20–3025.10)	2683.10 (1846.27–3448.75)	2862.45 (2500.67–3185.07)	2763.90 (2567.87–2965.80)	2564.75 (2150.45–3158.87)	2763.90 (2271.55–3020.75)
Carbohydrate (% energy)	68.80 (61.50–70.18)	68.53 (62.02–71.22)	50.10 (45.24–54.73)	47.91 (43.93–51.16)	55.83 (49.26–68.79)	52.13 (46.29–68.29)
Fat (% energy)	19.37 (16.71–25.61)	18.47 (15.47–25.35)	35.55 (31.86–38.23)	35.40 (33.80–38.53)	31.49 (19.37–35.86)	33.01 (18.56–36.91)
Protein (% energy)	12.45 (11.41–14.49)	13.12 (11.25–14.10)	14.12 (13.15–17.81)	15.86 (15.13–19.85)	13.42 (12.36–15.57)	14.97 (13.06–16.89)

Chinese group: before fasting vs. after fasting (CBF vs. CAF); Pakistani group: before fasting vs. after fasting (PBF vs. PAF); Total group: before fasting vs. after fasting (TBF vs. TAF), Significance levels: p-value was calculated; * < 0.05, ** < 0.01, by using Wilcoxon signed-rank test.

The average daily food intake and the respective energy ratios for ethnic groups are presented in Supplementary Table S1, Before fasting, Chinese participants had a significantly higher intake of grains, soy beans, and leaf vegetables with lower intake of livestock meat, fruits, and poultry compared with that of the Pakistani participants. After fasting, the Chinese group presented significantly higher intake of grains and soybeans but lower intake of milk, other vegetables, fruits, livestock meat, poultry, sweets, and seafood compared with the Pakistani group. We also found that the proportions of energy sources consumed by each group were significantly different between the Chinese and Pakistani groups, in that Chinese consumed significantly more CHO, while the Pakistani group consumed significantly more fats and proteins.

### Filtering and Sequencing of DNA

A total of 6,944,362 raw reads were derived from 68 fecal samples provided by 34 subjects. Low-quality reads (accounting for 9.94% of raw reads) were filtered, resulting in 6,254,015 high-quality reads with a median length of 415 bp. On average, we obtained 91,970 high-quality reads per sample (minimum: 45,129, maximum: 161,753, *SD*: 27,905) after processing.

### Comparative Taxonomic Analysis of Fecal Microbiota

To identify the types and abundance of dominant bacterial taxa among the total cohort, we determined the phylum and genus level taxonomic assignments of OTUs from each sample (Supplementary Figure S2). The results showed that the most dominant phylum across group was Firmicutes (44%), followed by Bacteroidetes (43%), and Proteobacteria (11%) (Supplementary Figure S3A). The predominant genera were *Prevotella* (18%), *Bacteroides* (14%), *Faecalibacterium* (5%), and *Dialister* (4%) (Supplementary Figure S3B).

#### Fasting Groups

**FIGURE 2:**
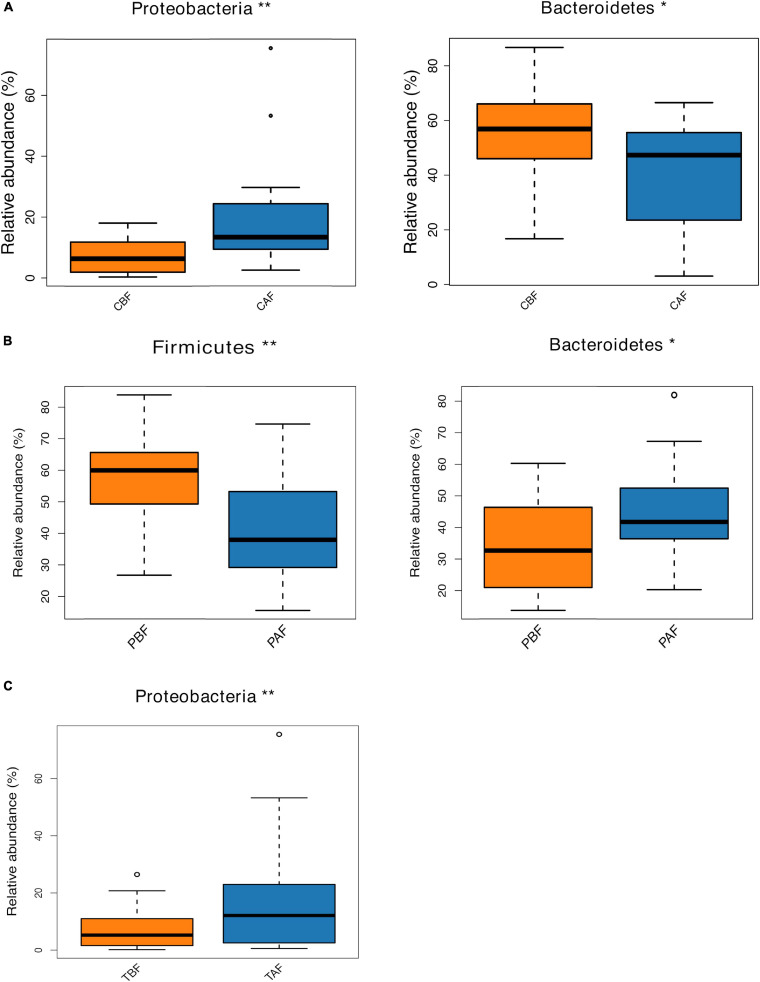
Relative abundances of bacterial phyla analyzed by using a metastats test was varied among each group at phylum level. **(A)** Chinese before fasting vs. Chinese after fasting, **(B)** Pakistani before fasting vs. Pakistani after fasting and **(C)** Total before fasting vs. Total after fasting; *P < 0.05, **P < 0.01.

**FIGURE 3:**
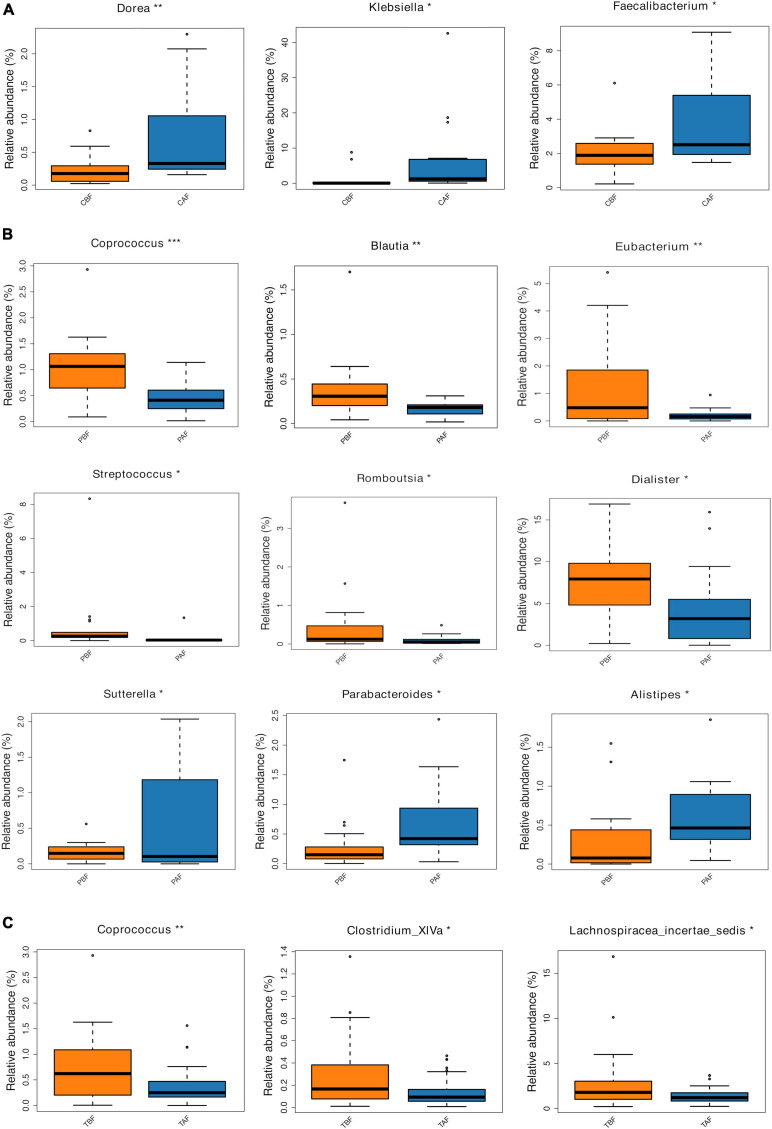
Relative abundances of bacterial taxa analyzed by using a metastats test was varied among each group at genus level. **(A)** Chinese before fasting vs. Chinese after fasting, **(B)** Pakistani before fasting vs. Pakistani after fasting, and **(C)** Total before fasting vs. Total after fasting; *P < 0.05, **P < 0.01, ***P < 0.001.

We next compared the prevalence of different taxa among fasting groups at the phylum and genus levels, and found a significant shift in these taxa before and after fasting for both ethnic groups. Specifically, within the Chinese cohort, the abundance of Bacteroidetes decreased, while Proteobacteria increased after fasting (CAF vs. CBF) (Figure 2A). Conversely, Bacteroidetes increased after fasting in the Pakistani group, and Firmicutes decreased (PAF vs. PBF) (Figure 2B). Comparisons among the full study cohort revealed a strongly significant increase in the abundance of Proteobacteria after fasting (TAF vs. TBF) (Figure 2C). At the genus level, *Dorea*, *Klebsiella*, and *Faecalibacterium* were all significantly enriched after fasting in comparisons between the CBF with CAF participants (Figure 3A). By contrast, *Sutterella*, *Parabacteroides*, and *Alistipes* were more abundant after fasting in the Pakistani group, while *Coprococcus*, *Blautia*, *Eubacterium*, *Streptococcus*, *Romboutsia*, and *Dialister* were more abundant before fasting (PAF vs. PBF) (Figure 3B). Notably, the shift in *Coprococcus* could be observed within the full cohort, since this genus, as well as *Clostridium_XlVa* and *Lachnospiracea incertae sedis* were all significantly more abundant prior to fasting in comparisons between fasting groups for total participants (TAF vs. TBF) (Figure 3C).

#### Ethnic Groups

We next compared differences in the predominant phyla between ethnic groups before and after fasting. The results showed that before fasting, the Pakistani group presented a significantly higher abundance of Firmicutes and Actinobacteria compared to the Chinese group, while the Chinese participants had higher levels Bacteroidetes (Supplementary Figure S4A). Phylum level comparisons after fasting between ethnic groups indicated that Lentisphaerae and Tenericutes were significantly more abundant in the Pakistani samples (Supplementary Figure S4B). At the genus level, Several genera were enriched in Pakistani and Chinese groups, respectively (Supplementary Figures S5A,B).

### Ethnicity Rather Than Fasting Drives Differences in Alpha Diversity

To account for differences in distribution (species richness) in addition to abundance, we used the Chao1, observed species, ACE, Coverage, Shannon, and Simpson indices to compare alpha diversity of gut microbiota between fasting groups (CBF vs. CAF, PBF vs. PAF, TBF vs. TAF, Supplementary Figure S6) and between ethnic groups (CBF vs. PBF, CAF vs. PAF, Supplementary Figure S7). First, comparisons between fasting groups showed that among Chinese participants, the ACE was higher after fasting, while the coverage index was lower after fasting (CBF vs. CAF; Supplementary Figure S6A); other indices showed no significant differences between fasting groups within either ethnic group (CBF vs. CAF, PBF vs. PAF, TBF vs. TAF). However, comparisons of alpha diversity between ethnic groups revealed significantly higher OTU abundance indices in the Pakistani group (i.e., observed OTUs, Chao1, and ACE) than in the Chinese group, while the coverage index was higher in the Chinese group than in the Pakistani group before fasting, suggesting greater evenness in the distribution of taxa before fasting. However, the Shannon and Simpson indices showed no differences between ethnic groups (CBF vs. PBF and CAF vs. PAF, Supplementary Figure S7). The alpha diversity showed that ethnicity rather than fasting drives differences in alpha diversity as Pakistani group presented significantly higher OTU abundance indices than Chinese group.

### Beta Diversity and Specific Taxa Vary Between Fasting Groups and Between Ethnic Groups

**FIGURE 4:**
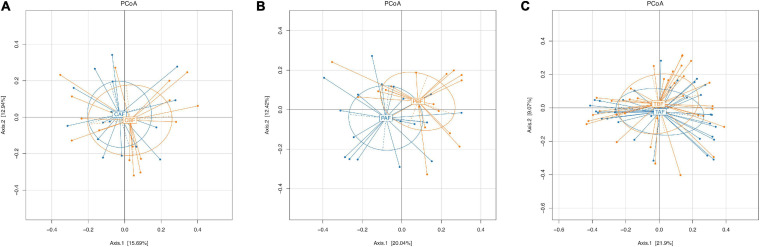
Principle coordinate analysis (PCoA) of the overall composition of the genera communities among the fasting groups. **(A)** Chinese before fasting vs. Chinese after fasting, **(B)** Pakistani before fasting vs. Pakistani after fasting, and **(C)** Total before fasting vs. Total after fasting groups. Each sample of respective groups were represented by a different colors symbol circles like CBF, PBF, TBF (orange circles), CAF, PAF, and TAF (blue circles). The percent of variation for each axis was explained and reported in square brackets using the Bray-Curtis.

To investigate differences in gut microbiota diversity between individual study subjects potentially attributable to ethnicity or fasting practices, we next performed PCoA, PERMANOVA test, Venn, and LEfSe analyses. To identify structural differences in gut microbiota all fasting group samples we conducted PCoA analysis using the Bray-Curtis model. This analysis showed that the microbial community composition shifted only slightly in Chinese fasting group after fasting (Figure 4A), whereas microbiota composition exhibited substantial divergence, with little overlap, between the before and after fasting groups of Pakistani participants (Figure 4B). This stability within the Chinese subjects before and after fasting was potentially reflected in the relative microbiota stability observed in comparisons between the total fasting groups (TAF vs. TBF) (Figure 4C). PCoA analysis comparing ethnic groups showed that ethnicity apparently drives substantial differences in microbiota structure, indicated by the lack of overlap between communities of different ethnicities both before (Supplementary Figure S8A) and after (Supplementary Figure S8B) fasting (CBF vs. PBF and CAF vs. PAF). Subsequent PERMANOVA tests further supported the significant differences between ethnic groups (CBF vs. PBF; CAF vs. PAF; *P* = 0.0001) and between the Pakistani fasting groups (PBF vs. PAF; *P* = 0.0129) (Supplementary Figure S9).

**FIGURE 5:**
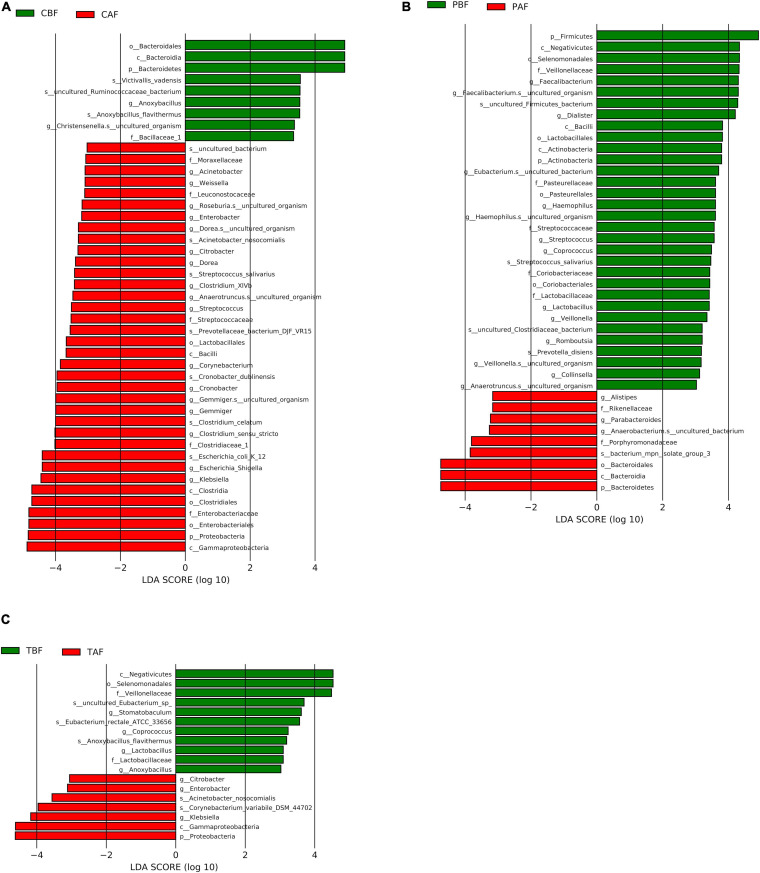
LEfSe analysis of signature taxa in the **(A)** Chinese before fasting vs. Chinese after fasting, **(B)** Pakistani before fasting vs. Pakistani after fasting, and **(C)** Total before fasting vs. Total after fasting groups. Linear discriminant analysis report represents the Prefixes abbreviations for the taxonomic rank of each taxon: phylum (p), class (c), order (o), family (f), genus (g), and species (s).

We then performed LEfSe analysis further explore the structure of microbiota and found that several specific taxa were significantly different in their distribution between fasting groups (LDA > 3, *P* < 0.05, Figure 5). In particular, Bacteroidetes were higher in abundance before fasting among Chinese participants (LDA = 4.85), while Proteobacteria were enriched after fasting (LDA = 4.79), possibly reflecting the significantly higher abundance of *Klebsiella* after fasting (LDA = 4.42). By contrast, Firmicutes were enriched before fasting among Pakistani subjects (LDA = 4.91), while Bacteroidetes were significantly higher in abundance after fasting (LDA = 4.74). At the genus level, these changes were indicated by the significant prevalence of *Dialister* and *Faecalibacterium* OTUs before fasting (LDA = 4.23 and 4.31, respectively). The substantial shifts observed in the Chinese group appeared to be reflected in the total cohort, since Proteobacteria (LDA = 4.69), and *Klebsiella* in particular (LDA = 4.18), were significantly more abundant after fasting in TBF vs. TAF comparisons. Interestingly, genus *Stomatobaculum* was significantly enriched in the TBF group (LDA = 3.63).

Further LEfSe analysis comparing ethnic groups showed the significant enrichment of several taxa (LDA > 3) (Supplementary Figures S10, S11). Before fasting, Firmicutes (LDA = 5.04), and especially *Prevotella* (LDA = 4.84) were found in high abundance among Pakistani subjects, and Bacteroidetes were significantly higher in the Chinese group (LDA = 5.03) potentially due to the high abundance of genus *Bacteroides* (LDA = 5.13). After fasting, Bacteroidetes remained dominant in the Chinese group (LDA = 4.98), while *Prevotella* and *Succinivibrio* were significantly enriched in the Pakistani group (LDA = 5.08 and 4.61, respectively).

**FIGURE 6:**
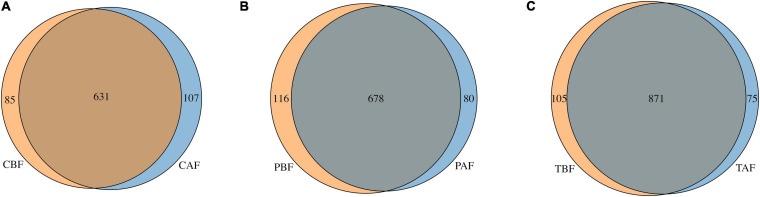
Venn diagram of unique and shared OTUs (operational taxonomic units) in different fasting groups. **(A)** Chinese before fasting vs. Chinese after fasting, **(B)** Pakistani before fasting vs. Pakistani after fasting and **(C)** Total before fasting vs. Total after fasting. The overlaps represent the common taxa between groups, and the non-overlapping portions represent unique taxa in each group.

Venn analysis showed that among the total 1,051 OTUs identified in all samples (Figure 6), 631 OTUs were shared between the Chinese before and after fasting groups, with 85 and 107 unique OTUs in the CBF and CAF groups, respectively. Similarly, 678 OTUs were shared between both Pakistani fasting groups, with 116 OTUs unique to the PBF group, and 80 OTUs unique to the PAF group. Venn comparisons of the total fasting groups showed that 871 OTUs were shared in between samples collected before and after fasting, with 105 OTUs unique to the TBF group and 75 OTUs unique to the TAF group. Venn analysis of ethnic differences showed that 534 OTUs were shared between the Chinese and Pakistani groups prior to fasting, while 182 were unique to the CBF group and 260 were unique to the PBF group (Supplementary Figure S12A). Similar overlap was observed in comparisons of ethnic groups after fasting, with 550 commonly shared OTUs, 188 unique to CAF, and 208 unique to PAF (Supplementary Figure S12B).

### Specific Dietary Components Are Significantly Correlated With Prevalent Taxa

**FIGURE 7:**
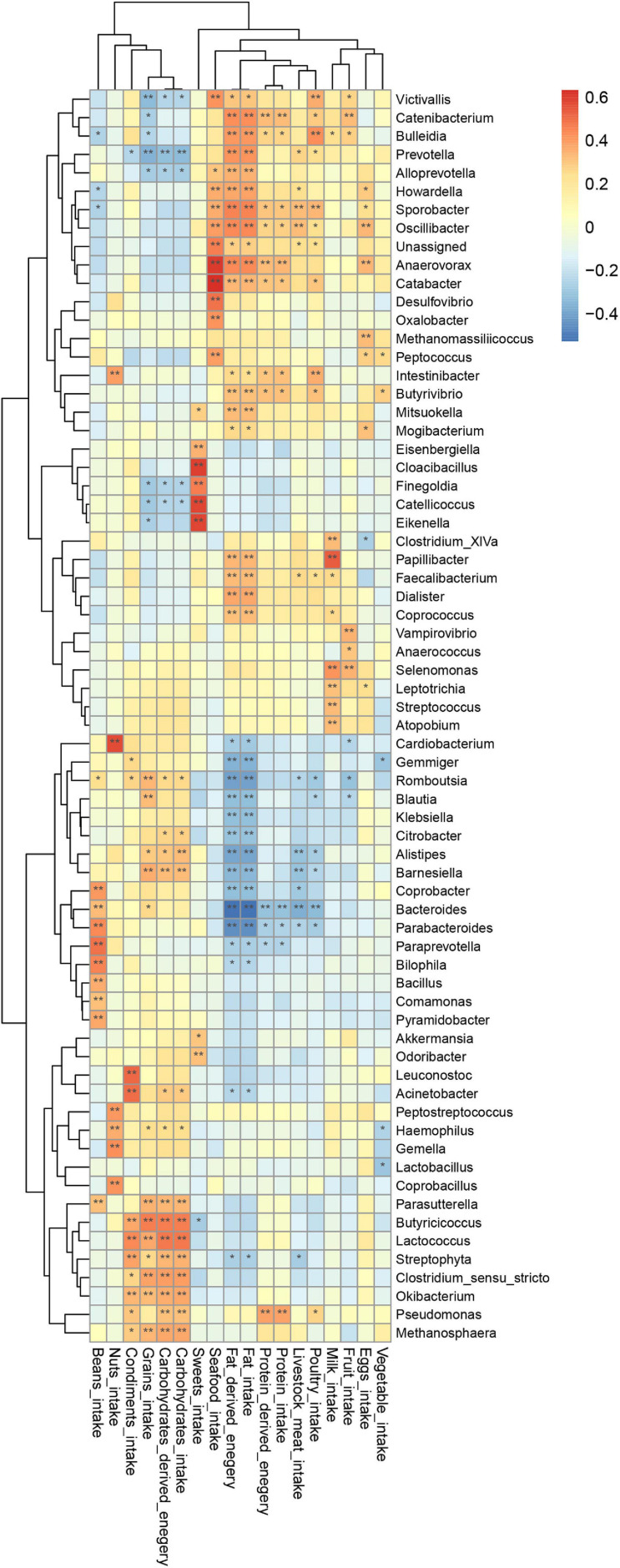
Correlation of genus with dietary intake; *p*-value was calculated using Pearson’s correlation; *P < 0.05, **P < 0.01.

To uncover whether latent relationships exist between the intake of specific nutrients sources and the composition of gut microbiota, we constructed a heatmap to illustrate correlations between bacterial taxa and diet. Pearson’s correlation test was used to examine the relationship between specific bacteria and respective nutrients (Figure 7).

This heatmap analysis showed that different microbial taxa were significantly associated with dietary intake, indicated by a Pearson’s correlation > 0.3 (Figure 5). In particular, CHO-derived energy was positively correlated with the abundance of *Alistipes*, *Barnesiella*, *Clostridium sensu stricto*, *Parasutterella*, *Butyricicoccus*, *Streptophyta*, *Lactococcus*, *Okibacterium*, *Pseudomonas*, and *Methanosphaera* (*r* = 0.31–0.46), but negatively correlated with *Prevotella* (*r* = −0.32). Fat-derived energy was positively associated with *Victivallis*, *Coprococcus*, *Butyrivibrio*, *Faecalibacterium*, *Mitsuokella*, *Papillibacter*, *Catabacter*, *Alloprevotella*, *Dialister*, *Howardella*, *Bulleidia*, *Catenibacterium*, *Oscillibacter*, *Anaerovorax*, *Sporobacter*, and *Prevotella* (*r* = 0.30–0.45), but was negatively associated with *Bacteroides*, *Parabacteroides*, *Romboutsia*, *Alistipes*, *Gemmiger*, *Barnesiella*, *Coprobacter*, *Klebsiella*, *Blautia*, and *Citrobacter* (*r* = −0.31 to (−0.52). Protein-derived energy was positively correlated with *Sporobacter*, *Intestinibacter*, *Catabacter*, *Butyrivibrio*, *Anaerovorax*, *Catenibacterium*, and *Pseudomonas* (*r* = 0.30–0.39), but negatively associated with *Bacteroides* (*r* = 0.31, *P* < 0.05). Notably, consumption of sweets was positively correlated with *Akkermansia*, *Odoribacter*, *Eisenbergiella*, *Catellicoccus*, *Eikenella*, *Cloacibacillus*, and *Finegoldi*a (*r* = 0.30–0.59).

## Discussion

In this study, we investigated whether fasting-associated dietary behavior affected shifts in gut microbiota composition among 34 healthy participants originating from China and Pakistan, using high-throughput 16S rRNA gene sequencing. To avoid any geographic bias, study participants from different ethnic groups were all recruited from the same city of Lanzhou (Gansu, China). Previous studies have reported that gut microbiota can be influenced by environment, host genetic background and ethnicity, drug intake, diet, and dietary behavior (Lloyd-Price et al., 2016; Gupta et al., 2017; Liao et al., 2018; Li et al., 2020; Liu et al., 2020). Zeb et al. (2020) reported that time-restricted feeding could regulate circadian rhythms associated with modulation of gut microbiota. However, among the changes in gut microbiota incurred by dietary behavioral patterns, the impact of fasting on gut microbiota has not been well investigated.

One previous study of the effects of fasting for Ramadan on gut microbiota revealed that fasting behaviors can lead to shifts in in gut microbial community composition (Ozkul et al., 2020). However, this study was unable to capture the influence of dietary composition on these shifts, only the practice of fasting, itself, thus leaving the effects of several, potentially major contributing factors unresolved. Furthermore, the inclusion of participants from two distinct ethnic groups can provide deeper insight into the drivers of gut microbiota dynamics.

### Intermittent Caloric Restriction by Fasting Likely Affects Gut Microbiota Composition and Beta Diversity

Dietary composition and behaviors have been shown to serve as strong influences that can shift gut microbiota structure (David et al., 2014; Mesnage et al., 2019). Since short-term alterations in dietary behavior, such as fasting, may alter the mealtimes and meal sizes, these behaviors may thus lead to rapid metabolic changes and consequent modifications in the proportions of Firmicutes and Bacteroidetes (Jumpertz et al., 2011; Li et al., 2017). At the phylum level, an increased proportion of Bacteroidetes and decreased Firmicutes were observed after fasting in the Pakistani group (Figure 2B), which is in agreement with previously described effects of caloric restriction on gut microbiota in a mouse model (Wang et al., 2018). Bacteroidetes were also significantly increased in the Chinese participants after fasting. This could be at least partially explained by enrichment for anaerobic fermenting taxa capable of degrading recalcitrant substrates and converting complex polysaccharides to simple sugars for ATP production (Dahiya et al., 2017). Shifts in the ratio of Firmicutes to Bacteroidetes has been previously described as part of a response to dietary changes, genetic and age variations in the host, and disease (Turnbaugh et al., 2006; Mariat et al., 2009; Jumpertz et al., 2011; Kwok et al., 2014; Lan et al., 2017). Our results indicate that fasting for Ramadan can also affect this ratio, suggesting that fasting can have implications on health. In addition, Proteobacteria were significantly enriched after fasting in the total group comparison (TBF vs. TAF), which is noteworthy because enrichment of Proteobacteria has been suggested as a gut microbial signature of dysbiosis (Shin et al., 2015).

In this study, we found that, at the genus level, *Sutterella* and *Parabacteroides* increased in abundance in the Pakistani group after fasting (Figure 3B). The prevalence of *Sutterella* suggested potential metabolic benefits, in light of previous reports indicating positive impacts by this genus on glucose levels, while *Parabacteroides* has been described as a potential factor associated with inhibition of weight gain (Zeng et al., 2019). Within the Chinese group, *Faecalibacterium* and *Butyricicoccus* increased after fasting, which aligned with the findings of Ozkul et al. (2020), suggesting that caloric restriction can enrich for beneficial bacteria (Ruiz et al., 2017). A study by Li et al. (2017) also found a higher abundance of *Butyricicoccus* after restricting food intake to simulate fasting in mice.

It warrants mention that in comparisons of total fasting groups, the taxa enriched prior to Ramadan fasting are primarily butyrate-producing bacteria, such as *Clostridium_XlVa* and *Lachnospiracea incertae sedis*, which can perform necessary functions in maintaining intestinal mucosa, and thus contribute to health (Peng et al., 2009; Louis et al., 2010). In addition, the significant enrichment of *Klebsiella* in the total cohort after fasting (TAF) suggests that gut health could also be negatively affected by fasting since the high abundance of *Klebsiella* has been associated with various diseases, such as neonatal necrotizing enterocolitis, multiple myeloma, and virus infections (Qin et al., 2015; Olm et al., 2019). However, we found significantly lower levels of genus *Coprococcus* after fasting in total group comparisons. This finding is also notable since previous studies have reported an association between high abundance of this genus and obesity (Kasai et al., 2015).

### Dietary Composition During Intermittent Caloric Restriction Can Significantly Contribute to Gut Microbiota

In the present study, Chinese and Pakistani participants self-reported the consumption of grains, vegetables, milk, livestock meat, poultry, sweets, beans, seafood, and fruits and the correlation between diet and gut microbiota showed that specific taxa were correlated with dietary intake, such as *Alloprevotella*, *Dialister*, and *Oscillibacter* with fat-derived energy (*r* = 0.37–0.44, *P* < 0.05), whereas other studies have reported a decreased of the *Alloprevotella* abundance in mice following a high calorie diet (Kong et al., 2019), while *Oscillibacter* and *Akkermansia* were negatively associated with obesity (Thingholm et al., 2019). Routy et al. (2018) studied patients with lung, renal, and other cancers, and found that patients with intestinal enrichment for *Akkermansia muciniphila* had better outcomes of immunotherapy. The results of other studies have suggested that *Akkermansia* can also help inhibit obesity and ameliorate alcohol-associated cirrhosis (Grander et al., 2018; Depommier et al., 2019). In our study, we observed that the consumption of sweets was positively correlated with the prevalence of *Akkermansia*. However, further study is required to clearly resolve how *Akkermansia* is affected by dietary intake.

Further PCoA analysis of dietary composition indicated that diets differed slightly across fasting groups, but dramatically differed (i.e., no overlap) across ethnic groups. This result was highly similar to the results of PCoA analysis for gut microbiota across the respective ethnic groups. Taken together, these findings highlight the clear contribution of diet on gut microbiota during fasting and across ethnic groups. Moreover, this study confirms the occurrence of shifts in gut microbial community triggered by dietary changes (e.g., including dietary habits such as fasting). Based on these results, we thus propose that ethnicity and fasting for Ramadan can alter the gut microbiota of humans, strongly mediated by changes in diet.

### The Effects of Ethnicity on Gut Microbiota Vary Between Pakistani and Chinese Groups

In agreement with other studies in humans, here we found that the dominant phyla in all groups were Firmicutes, Bacteroidetes, and Proteobacteria (Schnorr et al., 2014; Zhang et al., 2015). We observed that phylum-level structure was distinct among ethnic groups, with a higher abundance of Firmicutes in Pakistani participants than in Chinese before fasting, which instead had a higher prevalence of Bacteroidetes. Different relative proportions of Firmicutes and Bacteroidetes were previously reported by several studies, suggesting the potential influence of the host (Mariat et al., 2009; Kwok et al., 2014).

The *Faecalibacterium* and *Roseburia* genera belonging to Firmicutes phylum are well-established butyrate producers, which is necessary for maintaining intestinal mucosa (Peng et al., 2009; Louis et al., 2010). Metabolites such as butyrate, short-chain fatty acids, and acetate have been reported to improve gut barrier function (Peng et al., 2009), suppress the accumulation of insulin-mediated fat in adipose tissue (Kimura et al., 2013), and also contribute to prevention of colonic diseases in the host.

Schnorr et al. (2014) found significant changes in the structure of the gut community in comparisons of Italian and Hadza individuals. Despite the well-established, significant contribution of the ethnic background toward gut microbial alpha diversity (but not beta diversity) (Deschasaux et al., 2018), here we observed that both alpha and beta diversity were influenced by ethnicity independently of geographic location. This observation is possibly dependent on dietary intake and diet-related behavior. In particular, ethnic groups showed significant differences in several alpha diversity indices and clear separation in beta diversity. Pakistani group presented significantly higher OTU abundance indices (i.e., observed OTUs, Chao1, and ACE) than Chinese group, while the coverage index was higher in the Chinese group than in the Pakistani group before fasting. The alteration of alpha diversity among ethic groups were also reported by Liu et al. (2020), however beta diversity was not shifted according to ethnicity in their study. Future and ongoing studies will further clarify how ethnicity, which has many associated genetic, behavioral, and geographic factors, can contribute to driving the structure of microbiota in the context of regional diets and intermittent or stochastic changes in nutrient inputs.

### Strengths and Limitation of the Study

In the light of several other studies that provide evidence of differences in microbiota diversity and composition associated with distinct ethnic groups, and under the influences of nutritional intake and environment, here we endeavor to resolve the impact of dietary behavior (i.e., intermittent caloric restriction associated with Ramadan fasting) on gut microbiota by controlling geographic factors, and by addressing differences in diet and ethnic origin through the inclusion of participants from two distinct ethnic groups (Pakistani and Chinese) from the same city, socio-economic status, and presumably, similar environmental exposure.

Although the results of the study were significant but limited by the number of participants, it was necessary to further examined the influences of dietary behaviors such as fasting in a larger study cohort. Furthermore, future projects using high-throughput metabolomics, in conjunction with sequencing techniques, will provide remarkably higher resolution to our current understanding of the tripartite contributions of diet, microbiota, and their metabolites to human health.

## Conclusion

To our knowledge, this study provides the first investigation into the influence of Ramadan fasting on gut microbiota in either Chinese or Pakistani individuals. Our results demonstrate that, in the absence of geographic separation, significant differences exist in the structure, composition, and alpha- and beta diversities of gut microbiota in Chinese and Pakistani individuals, largely attributable to significant variations in diet. In addition, intermittent caloric restriction associated with fasting for Ramadan only appeared to affect beta diversity and the prevalence of some signature taxa. This work provides inroads to understanding how modification to dietary behavioral patterns can influence the generally diet-driven microbiota, whose further study can potentially guide behavior- or lifestyle-based treatments for targeted enrichment of specific taxa beneficial to gut health.

## Data Availability Statement

The datasets presented in this article are available with reasonable requests. Requests to access the datasets should be directed to XH, School of Public Health, Lanzhou University, Lanzhou China.

## Ethics Statement

The studies involving human participants were reviewed and approved by Medical Ethics Committee approval of the School of Public Health (GW-20171013), Lanzhou University, China. The patients/participants provided their written informed consent to participate in this study.

## Author Contributions

XH and RL conceived the study and designed the experiments. IkA and KL performed DNA extraction and drafted the manuscript. IkA, KL, SF, MH, and IzA coordinated in selecting field sampling sites and sample collection. IkA and KL analyzed the data and contributed to data interpretation. All authors contributed to the critical revision of the manuscript, read, and approved the final manuscript.

## Conflict of Interest

The authors declare that the research was conducted in the absence of any commercial or financial relationships that could be construed as a potential conflict of interest.

## Footnotes

1 http://www.ebi.ac.uk/ena/data/view/PRJEB38231
