# Supplementary material for: Ramadan Fasting Leads to Shifts in Human Gut Microbiota Structured by Dietary Composition
Source: Front Microbiol. 2021 Feb 18;12:642999. doi: 10.3389/fmicb.2021.642999 (PMC7930080; doi:10.3389/fmicb.2021.642999)
Supplement: Supplementary file 1 [file Data_Sheet_1.pdf]

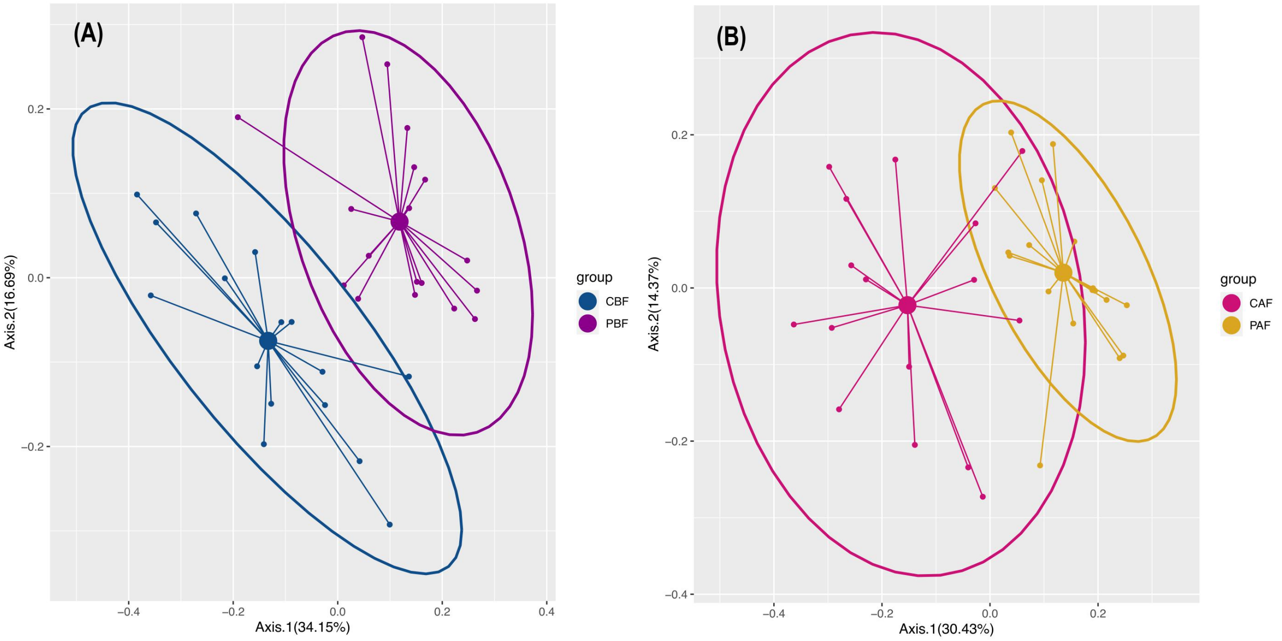

Figure S1. The nutrients intake profile by principal coordinate analysis (PCoA) of **(A)** Chinese before fasting versus Pakistani before fasting and **(B)** Chinese after fasting versus Pakistani after fasting groups. The variance presented by each component is written in brackets using Bray-Curtis.

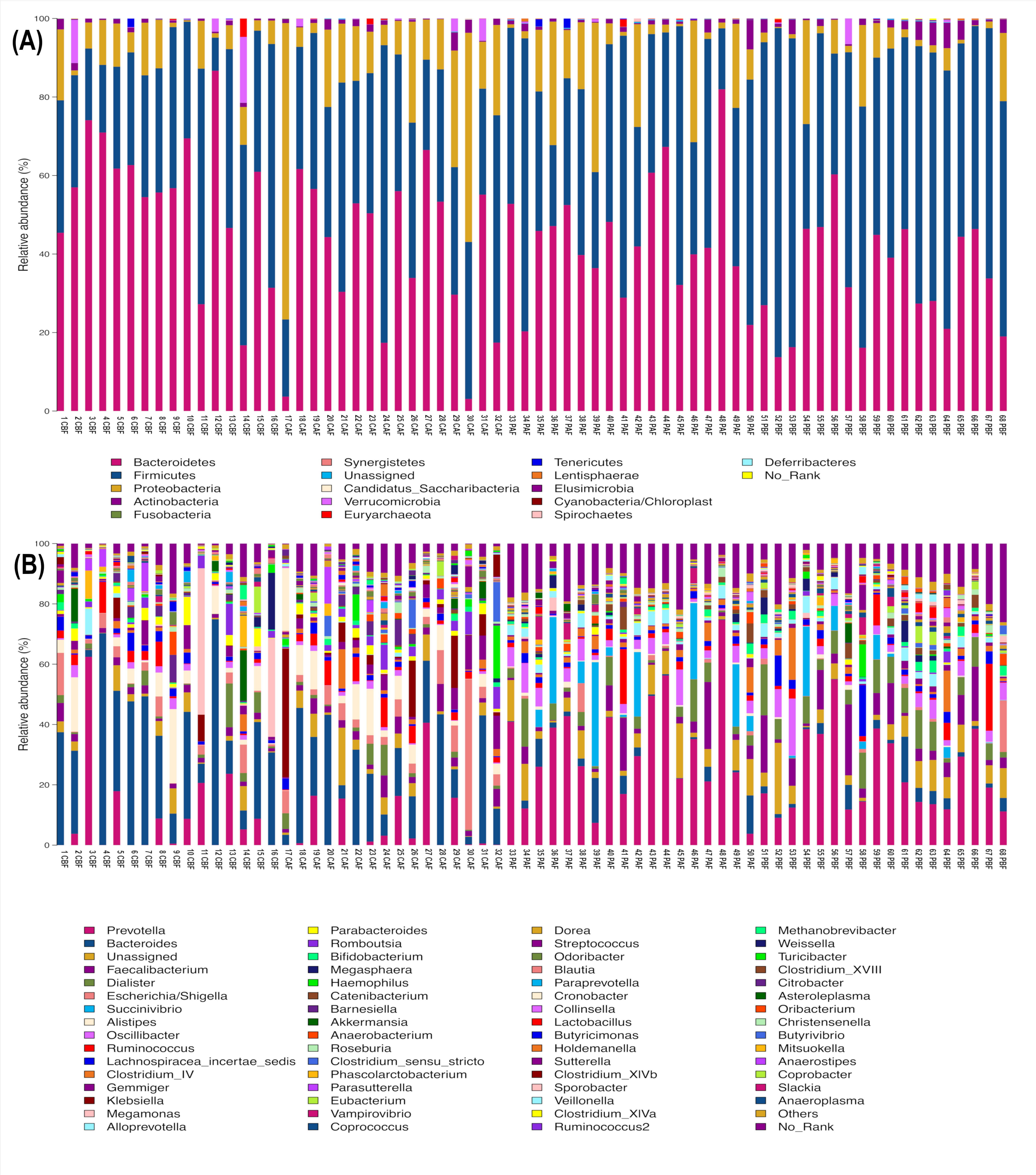

Figure S2. Individually taxonomic composition distribution in all fating and ethnic groups samples at level of **(A)** phylum **(B)** genus.

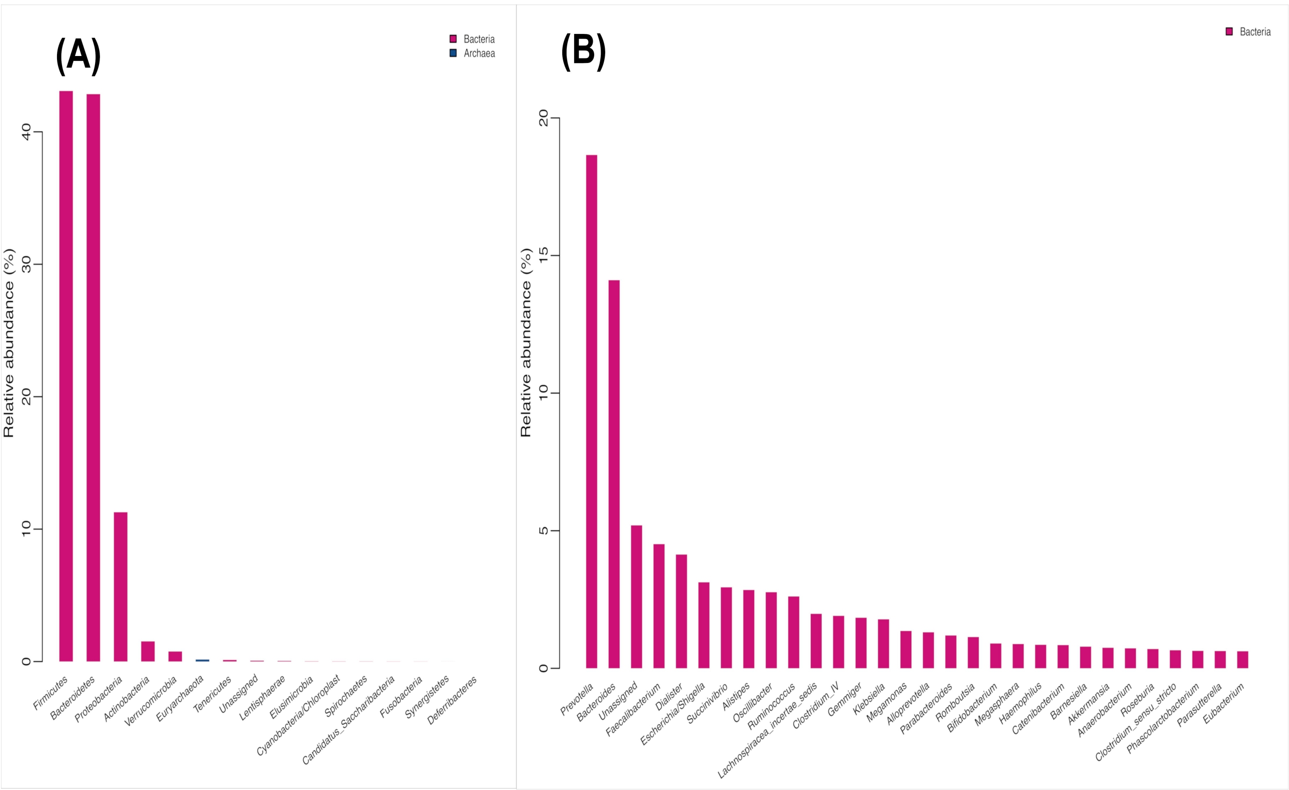

Figure S3. The relative abundance (%) of dominant phyla of all groups at the **(A)** phylum level and **(B)** genus level.

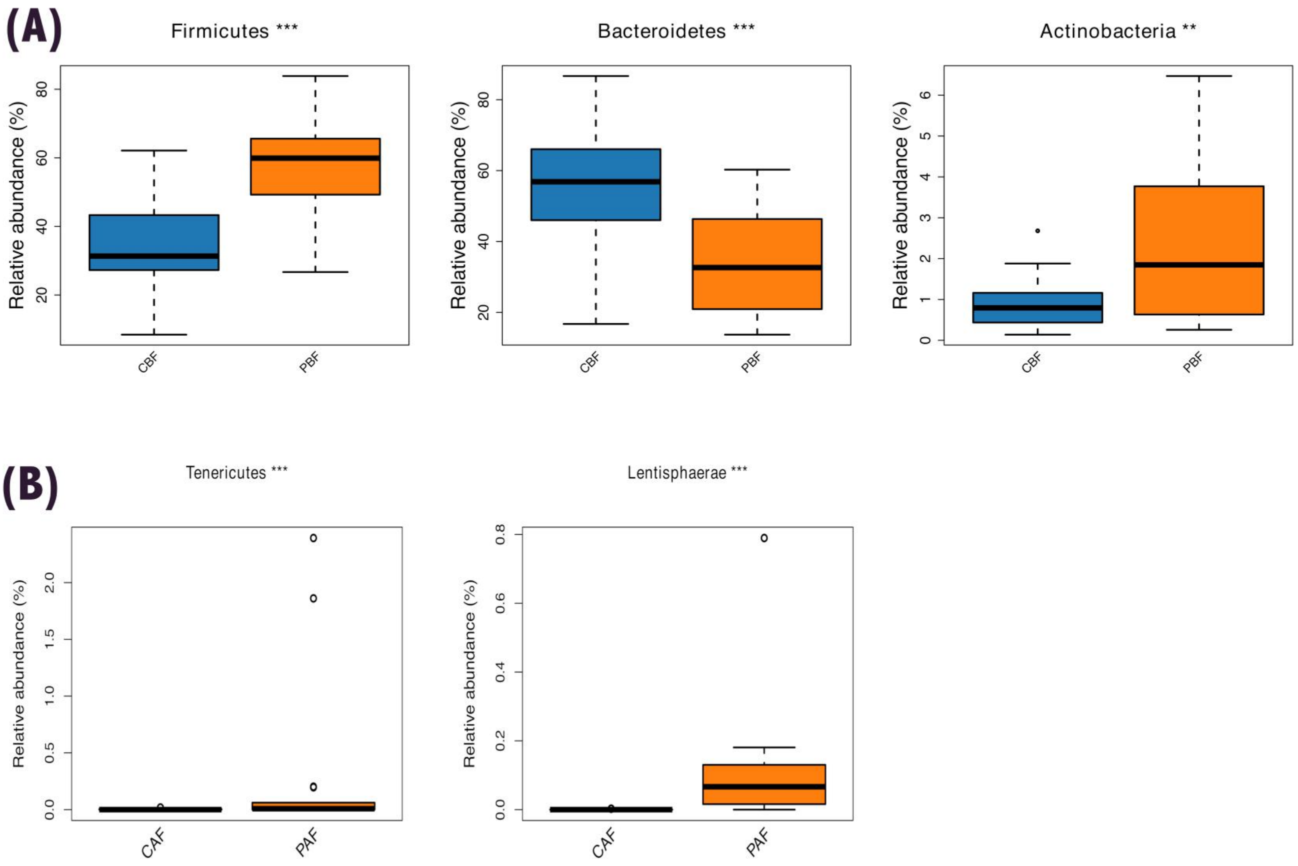

Figure S4. Relative abundances of bacterial phyla analyzed by using a metastats test was varied among each group at phylum level. **(A)** Chinese before fasting versus Pakistani before fasting and **(B)** Chinese after fasting versus Pakistani after fasting; *P<0.05, **P<0.01, ***P<0.001.

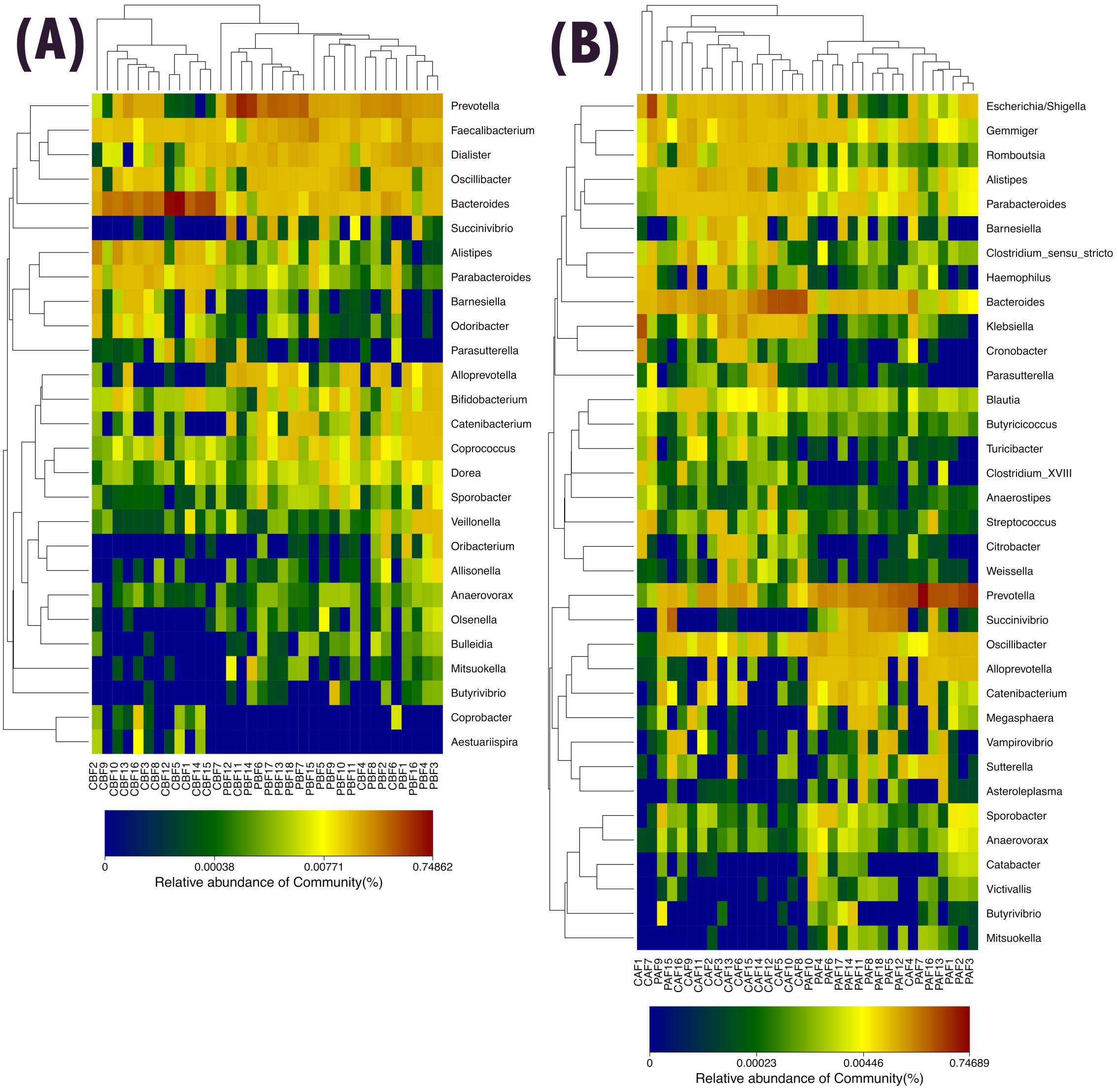

Figure S5. Relative abundances of bacterial taxa in percentage analyzed by using a metastats test was varied among each group at genus level. **(A)** Chinese before fasting versus Pakistani before fasting and **(B)** Chinese after fasting versus Pakistani after fasting; $P < 0.05$.

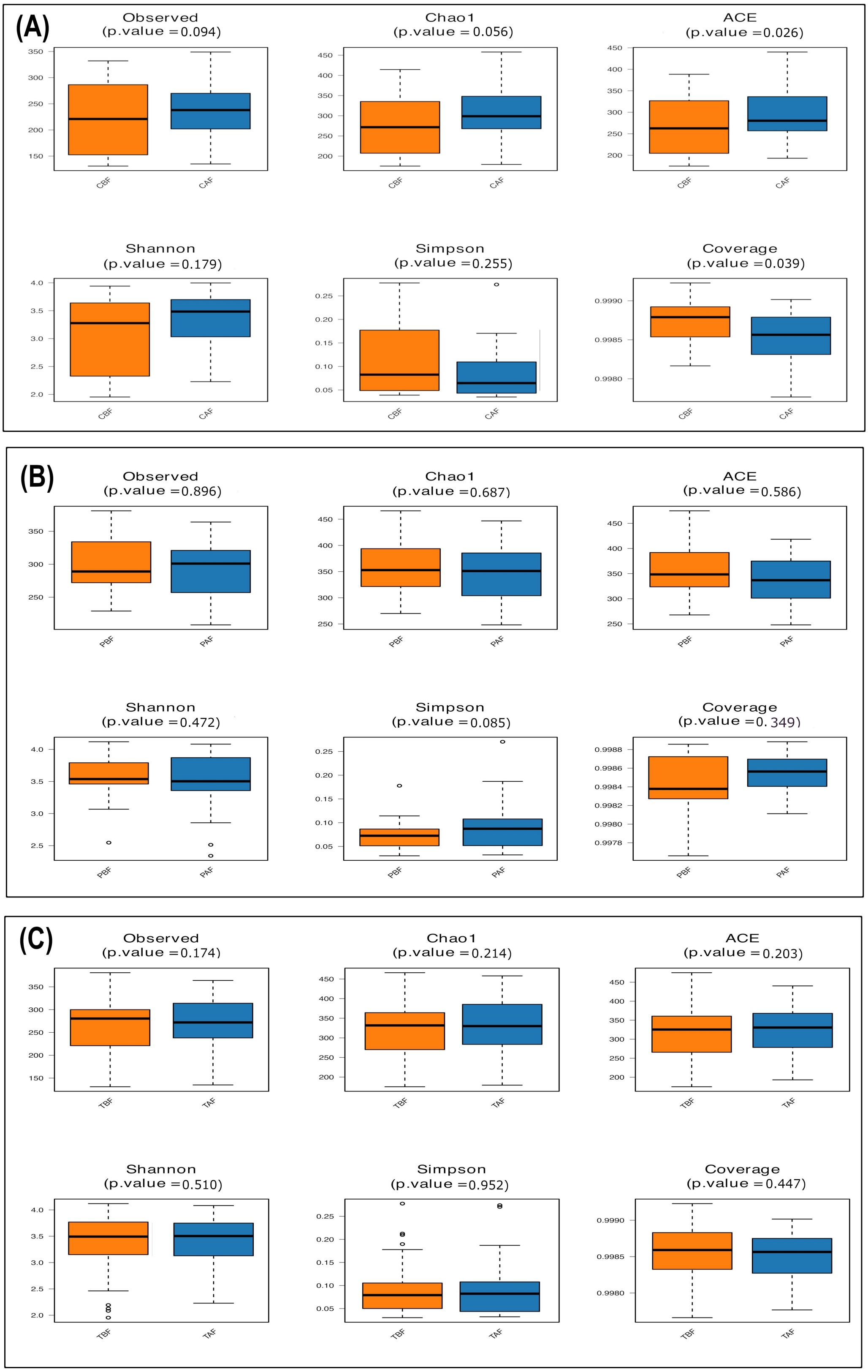

Figure S6. Alpha diversity of all Fasting indices, including community richness (observed species, chao, ace) and diversity (Shannon, Simpson, coverage) varied among each group**. (A)** Chinese before fasting versus Chinese after fasting, **(B)** Pakistani before fasting versus Pakistani after fasting and **(C)** Total before fasting versus Total after fasting groups; $P < 0.05$.

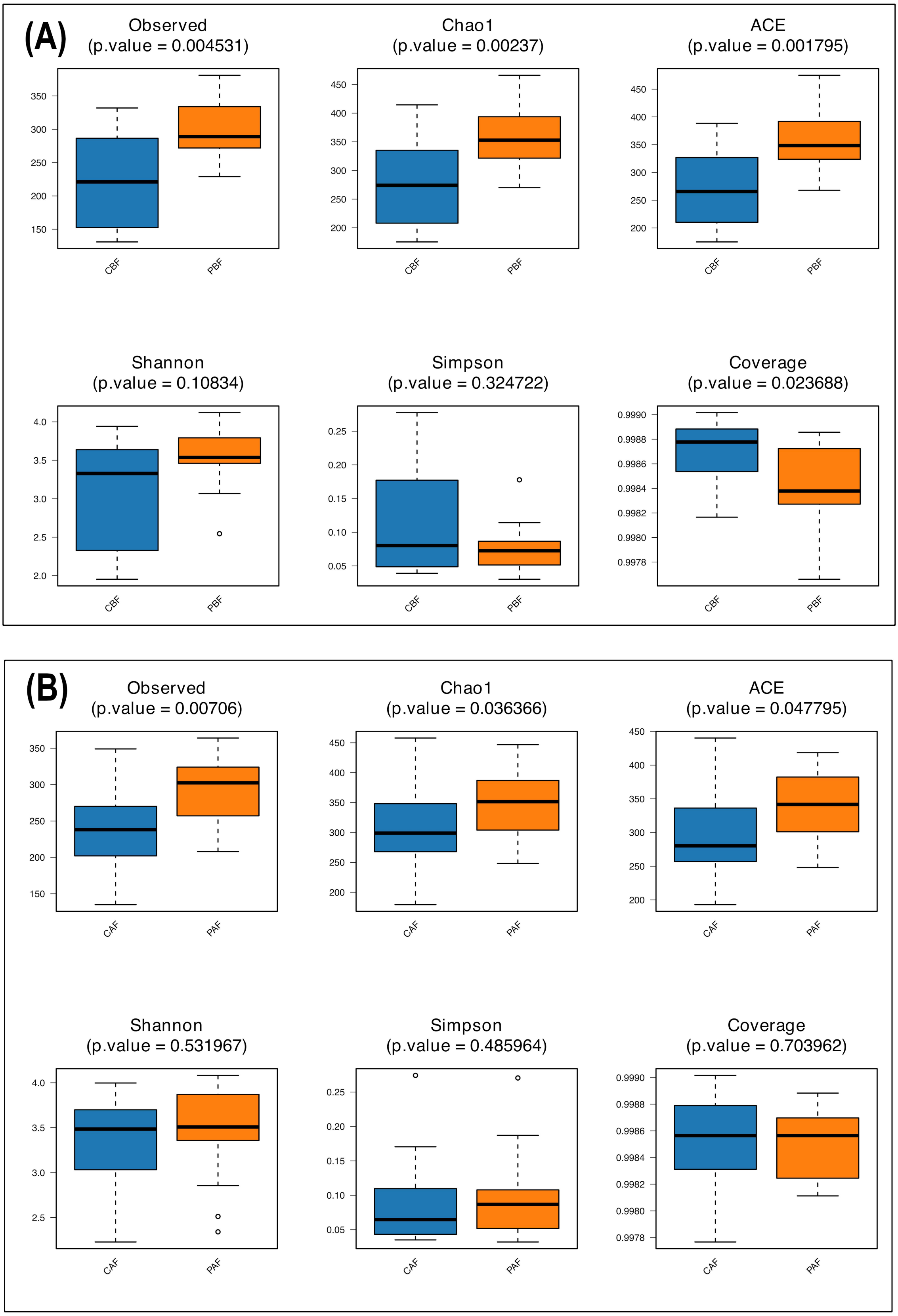

Figure S7. Alpha diversity of all ethnic indices, including community richness (observed species, chao, ace) and diversity (Shannon, Simpson, coverage) varied among each group**. (A)** Chinese before fasting versus Pakistani before fasting **(B)** Chinese after fasting versus Pakistani after fasting groups; $P < 0.05$.

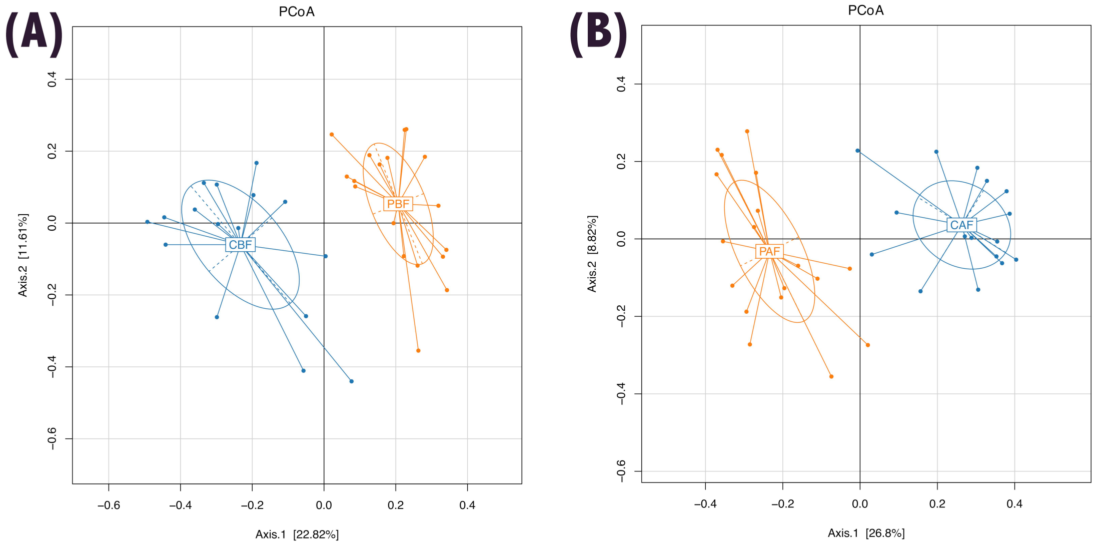

Figure S8. Principle coordinate analysis of the overall composition of the genera communities among the ethnic groups. **(A)** Chinese before fasting versus Pakistani before fasting and **(B)** Chinese after fasting versus Pakistani after fasting. Each sample of respective groups were represented by a different colours symbol circles like CBF, CAF (blue circles), PBF and PAF (orange circles). The percent of variation for each axis was explained and reported in square brackets by Bray-Curtis.

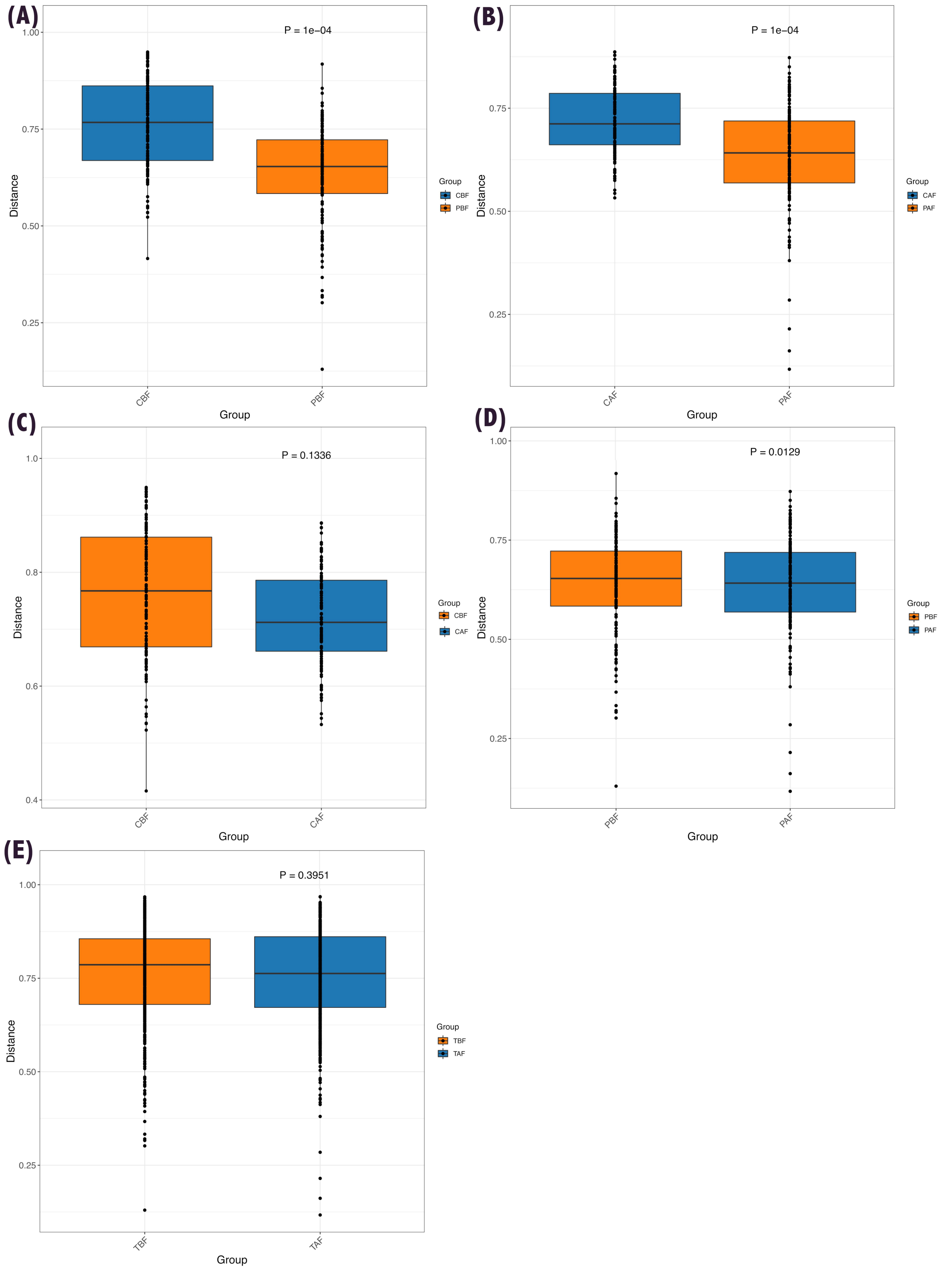

Figure S9. Permutational multivariate analysis of variance (PERMANOVA) among all paired of groups **(A)** Chinese before fasting versus Pakistani before fasting, **(B)** Chinese after fasting versus Pakistani after fasting, **(C)** Chinese before fasting versus Chinese after fasting, **(D)** Pakistani before fasting versus Pakistani after fasting and **(E)** Total before fasting versus Total after fasting groups; $P<0.05$.

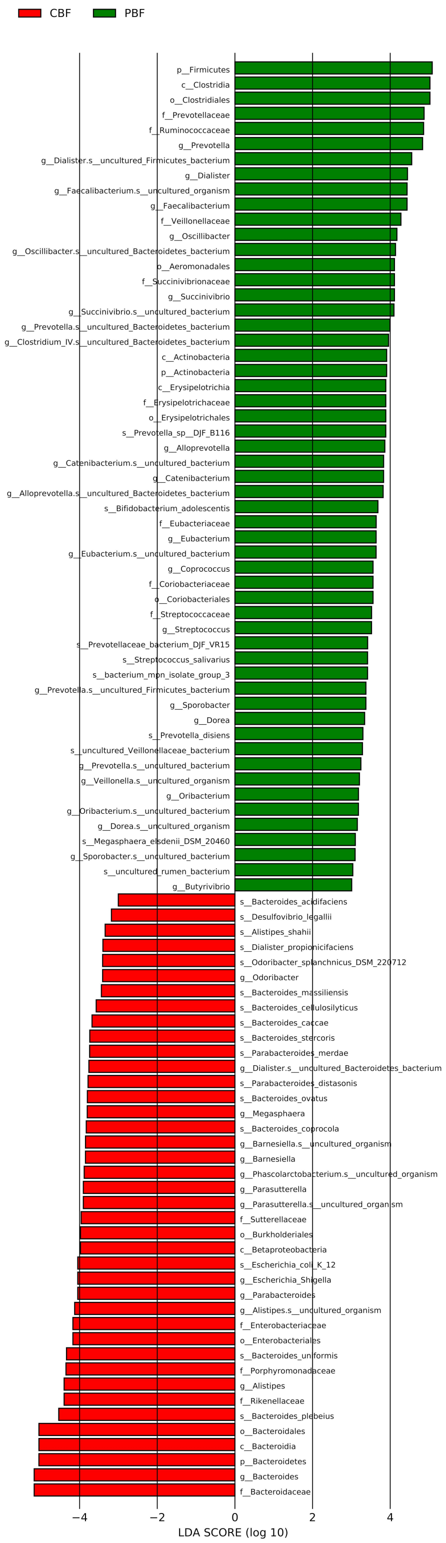

Figure S10. LEfSe analysis of signature taxa in the Chinese before fasting versus Pakistani before fasting groups. Linear discriminant analysis report represents the prefixes abbreviations for the taxonomic rank of each taxon: phylum (p), class (c), order (o), family (f), genus (g), and species (s).

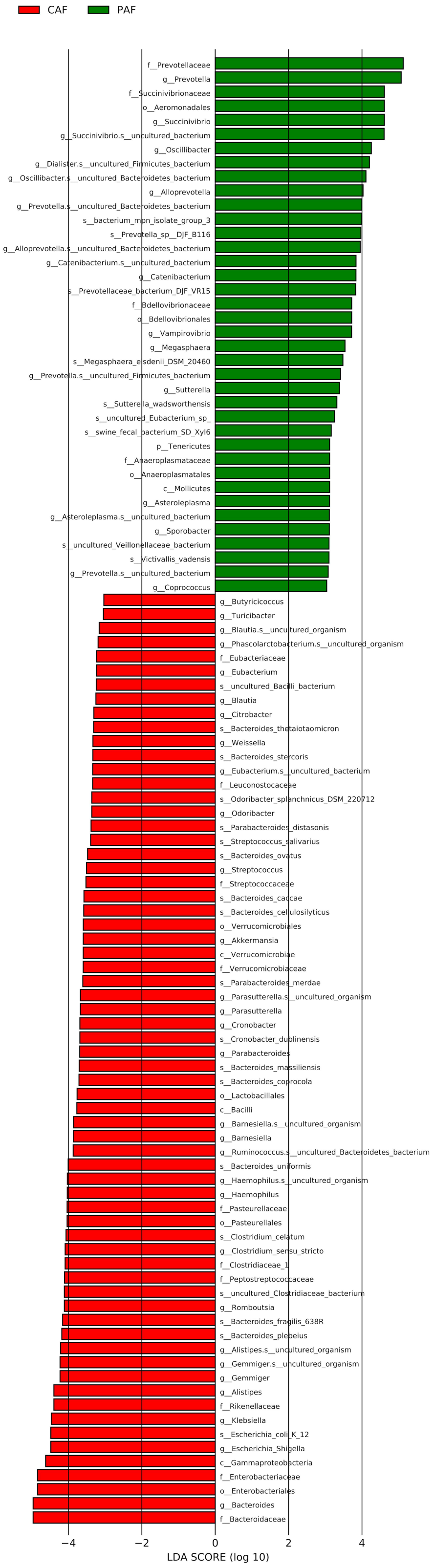

Figure S11. LEfSe analysis of signature taxa in the Chinese after fasting versus Pakistani after fasting groups. Linear discriminant analysis report represents the Prefixes abbreviations for the taxonomic rank of each taxon: phylum (p), class (c), order (o), family (f), genus (g), and species (s).

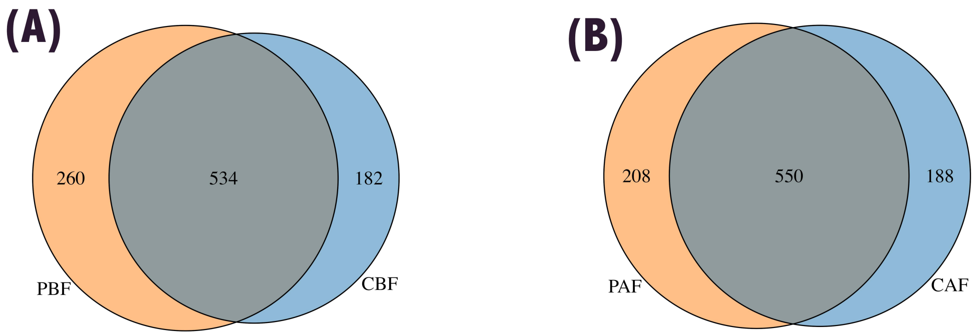

Figure S12. Venn diagram of unique and shared OTUs (operational taxonomic units) in different ethnic groups. **(A)** Chinese before fasting versus Pakistani before fasting and **(B)** Chinese after fasting versus Pakistani after fasting. The overlaps represent the common taxa between groups, and the non-overlapping portions represent unique taxa in each group.

Table S1. Average daily food intake, Total energy and energy ratios provided by macronutrients across ethnic groups.

Food components	Before fasting		After fasting	
	CBF	PBF	CAF	PAF
	n=16	n=18	n=16	n=18
Grains Intake (g)	735.01(537.35-921.35) *	580.35(487.33-666.68)	791.67(595.43-945.85) ***	466.69(333.35-533.35)
Milk Intake (g)	83.33(0.00-229.15)	151.71(82.00-437.58)	83.33(0.00-166.66)	161.66(129.09-208.34) *
Other vegetable Intake (g)	136.65(102.49-181.82)	76.66(74.20-207.92)	45.83(10.83-113.33)	197(132.91-332.48) ***
Fruit Intake (g)	0.00(0.00-82.92)	103(0.00-251.10) *	0.00(0.00-111.24)	216.16(120.82-304.16) **
Poultry Intake (g)	0.00(0.00-0.00)	153.33(79.16-244.99) ***	8.33(0.00-72.91)	158.33(83.33-354.15) ***
Livestock meat Intake (g)	19.16(10.11-87.83)	92.50(41.67-127.09) **	12.99(0.58-66.99)	80.00(41.25-127.49) **
Condiments Intake (g)	27.00(27.00-189.49)	56.00(55.75-163.34)	30.33(27.00-272.82)	55.00(55.00-109.99)
Egg Intake (g)	14.67(0.00-45.34)	31.83(0.00-65.67)	45.34(0.00-65.00)	0.00(0.00-65.33)
Soyabeans Intake (g)	106.66(27.91-226.67) ***	0.00(0.00-0.00)	12.50(0.00-33.34) **	0.00(0.00-0.00)
Sweets & cake Intake (g)	13.33(0.00-36.45	24.33(6.00-43.50)	0.00(0.00-19.16)	30.00(18.00-36.00) **
Leaf vegetables Intake (g)	16.67(0.00-43.64) **	0.00(0.00-0.00)	30.00(0.00-87.08)	0.00(0.00-24.00)
Seafood (g)	0.00(0.00-0.00)	0.00(0.00-0.00)	0.00(0.00-0.00)	0.00(0.00-133.34) **
Nuts Intake (g)	0.00(0.00-0.00)	0.00(0.00-0.00)	0.00(0.00-0.00)	0.00(0.00-0.00)
Total energy and energy ratios provided by macronutrients				
Energy (kcal)	2400.70(2025.20-3025.10)	2862.45(2500.67-3185.07)	2683.10(1846.27-3448.75)	2763.90(2567.87-2965.80)
Carbohydrate (% energy)	68.80(61.50-70.18) ***	50.10(45.24-54.73)	68.53(62.02-71.22) ***	47.91(43.93-51.16)
Fat (% energy)	19.37(16.71-25.61)	35.55(31.86-38.23) ***	18.47(15.47-25.35)	35.40(33.80-38.53) ***
Protein (% energy)	12.45(11.41-14.49)	14.12(13.15-17.81) **	13.12(11.25-14.10)	15.86(15.13-19.85) **

Before fasting; Chinese versus Pakistani (CBF vs PBF); After fasting: Chinese versus Pakistani (CAF vs PAF); Significance levels: p-value was calculated; * < 0.05, ** < 0.01, *** < 0.001, by using Mann-Whitney U test.
